# Investigating the Spatial Biases and Temporal Trends in Insect Pollinator Occurrence Data on GBIF

**DOI:** 10.3390/insects16080769

**Published:** 2025-07-26

**Authors:** Ehsan Rahimi, Chuleui Jung

**Affiliations:** 1 Agricultural Research Institute, GyeongKuk National University, Andong 36729, Republic of Korea; ehsanrahimi666@gmail.com; 2 Department of Plant Medical, GyeongKuk National University, Andong 36729, Republic of Korea

**Keywords:** GBIF, species distribution models, pollinators, bees, butterflies, hoverflies

## Abstract

Research in biogeography, ecology, and biodiversity hinges on the availability of comprehensive datasets that detail species distributions and environmental conditions. At the forefront of this endeavor is the Global Biodiversity Information Facility (GBIF). This study focuses on investigating spatial biases and temporal trends in insect pollinator occurrence data within the GBIF dataset, specifically focusing on three pivotal pollinator groups: bees, hoverflies, and butterflies. Addressing these gaps in GBIF data is essential for comprehensive analyses and informed pollinator conservation efforts. We obtained occurrence data from GBIF for seven bee families, six butterfly families, and the Syrphidae family of hoverflies in 2024. Spatial biases were addressed by eliminating duplicate records with identical latitude and longitude coordinates. Species richness was assessed for each family and country. Temporal trends were examined by tallying annual occurrence records for each pollinator family, and the diversity of data sources within GBIF was evaluated by quantifying unique data publishers. We identified initial occurrence counts of 4,922,390 for bees, 1,703,131 for hoverflies, and 31,700,696 for butterflies, with a substantial portion containing duplicate records. On average, 81.4% of bee data, 77.2% of hoverfly data, and 65.4% of butterfly data were removed post-duplicate elimination for dataset refinement. Our dataset encompassed 9286 unique bee species, 2574 hoverfly species, and 17,895 butterfly species. Our temporal analysis revealed a notable trend in data recording, with 80% of bee and butterfly data collected after 2022, and a similar threshold for hoverflies reached after 2023. The United States, Germany, the United Kingdom, and Sweden consistently emerged as the top countries for occurrence data across all three groups. The analysis of data publishers highlighted iNaturalist.org as a top contributor to bee data. Overall, we uncovered significant biases in the occurrence data of pollinators from GBIF. These biases pose substantial challenges for future research on pollinator ecology and biodiversity conservation.

## Simple Summary

This study investigates spatial biases and temporal trends in insect pollinator occurrence data from the Global Biodiversity Information Facility (GBIF), focusing on bees, hoverflies, and butterflies. Occurrence data for these groups were obtained in 2024, with duplicates removed to refine the dataset. Species richness was assessed by family and country, and temporal trends were analyzed by examining annual records. The dataset included 9286 bee species, 2574 hoverfly species, and 17,895 butterfly species, with significant data collection occurring after 2022 for bees and butterflies, and after 2023 for hoverflies. The United States, Germany, the United Kingdom, and Sweden were the top contributors of occurrence data. iNaturalist.org emerged as a major data publisher for bees. This study highlights substantial spatial and temporal biases in GBIF pollinator data, which could hinder future research and conservation efforts. Addressing these biases is crucial for improving the reliability of pollinator ecology and biodiversity studies.

## 1. Introduction

Pollinators are essential to both natural ecosystems and agriculture, facilitating the reproduction of over 90% of flowering plant species and contributing up to 40% of the nutrients in the human diet [1,2]. Insect pollination alone contributes 9.5% to the global economic value of crops [3], with animal pollination services estimated to be worth between USD 195 and USD 387 billion annually [4]. Countries such as Britain, Germany, and Japan are particularly vulnerable to economic losses resulting from pollinator decline [5]. Pollinating insects encompass various groups such as moths, butterflies, bumblebees, honeybees, solitary bees, and hoverflies. Among these, bees are of exceptional importance, pollinating approximately 35% of the world’s food production [6]. As ectothermic organisms, bees are highly sensitive to temperature, which influences their foraging activity and ecological function. Notably, honeybees and bumblebees visit over 90% of major food crops globally [2], underscoring their central role in food security and ecosystem health.

Understanding the distribution and trends of pollinators relies heavily on species occurrence data. The Global Biodiversity Information Facility (GBIF) serves as the world’s largest open-access database for biodiversity records, aggregating digitized data from museum collections, field surveys, and citizen science initiatives. As of 2025, GBIF includes over 3 billion occurrence records from 1,148,235 datasets contributed by 2504 institutions, and has been cited in more than 13,000 scientific publications (https://www.gbif.org/, accessed on 30 June 2025). GBIF has become an indispensable tool in biogeography, ecology, and conservation research, enabling global-scale analyses of species distributions, including those of pollinators [7,8,9].

Despite its value, GBIF data suffer from multiple biases and quality issues that limit their utility. Spatial and taxonomic biases result from uneven sampling efforts, disparities in national funding and data-sharing practices, and inconsistent digitization [8,10,11,12,13]. Garcia Garcia-Rosello et al. [14] showed that 34% of terrestrial grid cells worldwide lack any GBIF insect data, and less than 0.5% are highly complete. Troudet et al. [15] found that birds are overrepresented while insects are significantly underrepresented in GBIF. Additional limitations include inconsistent metadata, duplicated records, and poor alignment with species’ actual ranges [16,17,18,19]. Currently, insects make up less than 10% of all GBIF records, and butterfly data alone account for nearly half of this subset [19].

This study investigates GBIF occurrence data for three ecologically important pollinator groups: bees, butterflies, and hoverflies. We aim to evaluate data completeness, identify spatial and temporal patterns, assess taxonomic and geographic biases, and quantify duplication. By producing cleaned and standardized datasets for each group, our goal is to support more accurate modeling and monitoring of pollinator biodiversity. This work complements ongoing efforts such as the Database of Pollinator Interactions (DoPI) [20], the BeeBDC R package 1.2.1 [21], regional datasets from Chile [22], and other global platforms like Web of Life and IWDB. Ultimately, this study helps bridge data gaps and provides a valuable open-access resource for researchers and conservation practitioners [23].

## 2. Methodology

### 2.1. GBIF Dataset for Pollinators

For this study, we retrieved occurrence data for pollinator species from the GBIF, a globally recognized open-access platform that compiles species distribution records from a wide variety of data providers. GBIF allows users to search and download biodiversity data at multiple taxonomic levels, including species, genus, family, and order. To gather relevant data for our analysis, we searched GBIF using the family names of each pollinator group studied. For each family, GBIF compiles all associated species records and offers them as downloadable ZIP archives, including a file named occurrence.txt containing detailed information about each record.

Each occurrence.txt file can include up to 221 columns, offering rich metadata about taxonomic classification, observation context, geographic coordinates, and dataset source. However, many of these columns are either sparsely populated or irrelevant for our study objectives. As such, we focused only on a subset of essential fields that are critical for spatial and ecological analysis. These include the scientific name (*scientificName*), country (country or *countryCode*), geographic coordinates (*decimalLatitude*, *decimalLongitude*), observation date (*eventDate*), occurrence status (*occurrenceStatus*), basis of record (*basisOfRecord*), dataset name (*datasetName*), and key taxonomic levels such as kingdom, phylum, class, order, family, genus, and species.

The *occurrenceStatus* column plays a key role in distinguishing whether a species was reported as *present* or *absent* at a specific location. Since GBIF absence data can often be inferred or uncertain—rather than empirically recorded—we included only presence records in our analysis. This decision reduces the risk of incorporating misleading or incomplete data and ensures a more accurate reflection of observed species distributions.

Another important filter was applied based on the *basisOfRecord* field, which describes the origin of each observation. GBIF classifies records into several types, such as human observation, machine observation, material sample, material citation, preserved specimen, fossil specimen, living specimen, and general occurrence. To ensure consistency and reliability, we included only human observation records—those directly reported by individuals in the field—since they align most closely with the type of data needed for ecological modeling and are less prone to certain types of systematic error.

The *datasetName* column identifies the specific dataset from which each record originates. GBIF integrates data from a wide range of sources, including citizen science platforms, museum collections, and institutional biodiversity surveys. Notable examples include *iNaturalist Research-grade Observations*, *Observation.org: Nature data from around the world*, *BeeWalk: Bumblebee distributions for Great Britain (2008–2022)*, *Naturalis Biodiversity Center (NL)—Hymenoptera*, and the *Snow Entomological Museum Collection*, among many others. These datasets differ in scope and quality but collectively contribute to the extensive and diverse repository that GBIF offers.

Spatial information is central to our study, and we relied on the *decimalLatitude* and *decimalLongitude* fields to extract the precise location of each observation. However, many GBIF records are incomplete or lack valid coordinates. To maintain spatial accuracy and analytical integrity, we excluded all records without valid geographic coordinates from our dataset. Accordingly, we obtained occurrence.txt files from GBIF [24,25,26,27,28,29] in 2024 for seven bee families, six butterfly families, and the Syrphidae family of hoverflies. These files contained the key information described above and served as the foundation for further analyses conducted in this study.

### 2.2. Spatial Bias Analysis

To assess potential spatial biases in our dataset, we first processed the GBIF occurrence data using R software 4.3.1. For each pollinator family—bees, butterflies, and hoverflies—we read the corresponding occurrence.txt file using the *read.delim()* function, specifying tab delimiters and ensuring proper parsing of character and numeric fields. These data files were read into data frames for further manipulation and spatial filtering.

To identify and eliminate duplicate spatial records, we used the latitude and longitude values provided in the *decimalLatitude* and *decimalLongitude* columns. Duplicate records were defined as those with identical coordinate pairs, regardless of other attributes. In R, we applied the *duplicated()* function on a combination of latitude and longitude columns using the dplyr package [30], specifically the command *distinct()* or *!duplicated()* on *paste(decimalLatitude, decimalLongitude).* This ensured that only one occurrence per unique spatial point was retained for each family, minimizing potential overrepresentation of frequently sampled locations such as research stations or biodiversity hotspots.

To analyze the global distribution of these cleaned occurrence points, we overlapped the spatial data with a world country boundary map. For this purpose, we used the *rnaturalearth* R package [31], which provides freely available high-quality vector maps. Specifically, we used the function *ne_countries(scale = “medium”, returnclass = “sf”)* to retrieve a medium-resolution world map in Simple Features (sf) format. This map was used as a geographic reference layer to determine the country in which each occurrence point fell and to remove records falling outside terrestrial landmasses.

Using the sf package, we converted the occurrence data frames to spatial objects via the *st_as_sf()* function, specifying coordinates and the appropriate coordinate reference system (typically WGS84, EPSG:4326). We then applied a spatial join using the *st_join()* or *st_intersects()* functions to associate each occurrence point with its corresponding country polygon. Through this operation, we were able to extract the country name for each point using the attributes of the intersecting country polygon.

Points that failed to intersect with any land polygon—typically those located in the ocean due to erroneous coordinates—were identified as NA after the join. These points were then excluded from the dataset using filter(!is.na(country)) or similar logic. As a result, only records with valid land-based geographic coordinates associated with identifiable countries were retained for downstream analysis. Finally, we used the dplyr package to count the number of valid presence points per country for each family using functions such as *group_by(country)* and *summarise(n = n()).*

### 2.3. Pollinator Occurrence Data

For each pollinator group analyzed in this study—including bees, butterflies, and hoverflies—we aimed to evaluate the global distribution and sampling density of occurrence records. To achieve this, we created a global raster grid based on the WGS84 geographic coordinate system, with a spatial resolution of 1 degree in both latitude and longitude. This resolution provided a standardized spatial framework for summarizing occurrence data across the entire terrestrial surface of the Earth.

As we already had the geographic coordinates (*decimalLatitude* and *decimalLongitude*) for each occurrence record within each pollinator family, we used these values to assign points to spatial grid cells. An empty global raster template was first created using the *raster()* function from the raster package, with the extent set to cover the entire globe and a resolution of 1 degree. This empty raster served as the base structure for spatial aggregation.

We then converted the point occurrence data into spatial objects using the *st_as_sf()* function from the sf package, which allowed for integration with spatial operations. To populate the raster grid, we used the *rasterize()* function from the raster package, specifying the counting function (*fun = “count”*) so that each cell would represent the number of occurrence records falling within it. Cells that contained no records were automatically assigned a value of zero using the background parameter.

The output was a raster layer in which each cell represented the total number of presence points recorded for a given pollinator group within that 1-degree area. This allowed us to identify spatial hotspots of sampling effort, as well as regions where data were sparse or absent. By using a consistent global grid, we were able to directly compare the spatial coverage and data density of different pollinator families.

To further analyze and visualize the results, we used functions such as *plot()* and *tm_shape()/tm_raster()* from the tmap package [32] to generate global maps of pollinator occurrence density. Additionally, we summarized data per country using *st_join()* (from sf) to intersect point data with country polygons (retrieved using *ne_countries()* from the rnaturalearth package), followed by aggregation with *group_by()* and *summarise()* from the dplyr package.

### 2.4. Pollinator Richness Data

To assess the species richness of the three major pollinator groups considered in this study—bees, butterflies, and hoverflies—we calculated the number of unique species represented in the occurrence data for each group. This measure of richness allowed us to evaluate both the taxonomic diversity of each group and its spatial distribution at the country level.

We began with the cleaned GBIF occurrence datasets for each group, which included a column labeled *scientificName*, containing the full binomial name of each observed species. This information was already available in the downloaded occurrence.txt files. Since GBIF records are structured by taxonomic hierarchy (including family, genus, and species), we were able to filter and process the records group by group. Using the *filter()* function from the dplyr package, we isolated records belonging to each target family within bees, butterflies, and hoverflies. We then used the *distinct()* function to extract unique combinations of species names and families, allowing us to calculate the species richness per family by applying *n_distinct*(*scientificName*).

To assess species richness at the country level, we relied on the *countryCode* or country field provided in the occurrence data, which indicates the country in which each record was observed. For each group, we grouped the records by country using the *group_by*(*country*) function and then calculated the number of unique species per country using *summarise(richness = n_distinct(scientificName)).* Before performing the country-level analysis, we ensured that all occurrence records had valid and non-missing country identifiers. For records that did not include a country or *countryCode*, we spatially joined the coordinate data to a world country polygon *shapefile* using the *st_join()* function from the sf package, with polygons retrieved via the *ne_countries()* function from the rnaturalearth package. This step allowed us to assign countries based on the location of each point, improving completeness and spatial accuracy.

### 2.5. Temporal Trends Data

To analyze temporal trends in pollinator occurrence data, we quantified the number of records for each pollinator family on an annual basis. The primary temporal information was obtained from the *eventDate* field within the GBIF occurrence datasets, which records the date when each pollinator observation was made. Since this field often contains full date strings, we first extracted the year component from each *eventDate* entry to enable aggregation at the yearly scale. This was accomplished using date handling functions from the lubridate package in R, such as *year()* to parse and extract the year from date-time strings.

After converting the full date into a year, we filtered out records with missing or incomplete date information to ensure that the temporal data used in the analysis were reliable. We then grouped the occurrence data by pollinator family and by year using the *group_by()* function from the dplyr package. Within each group, we used the *summarise()* function to count the total number of occurrence records per year, thereby generating a time series of annual sampling effort for each pollinator family.

### 2.6. Publisher Information

To evaluate the diversity of data sources contributing to the pollinator occurrence records, we counted the number of unique publishers for each pollinator family. Publisher information was extracted from the *publishingOrg* or publisher fields within the GBIF occurrence datasets that identify the institutions, research groups, or organizations responsible for submitting each record to GBIF. These fields provide valuable metadata about the origin and breadth of data sources, helping to assess the extent of collaboration and dataset diversity across taxonomic groups.

Using R, we first ensured that the publisher fields were clean and consistently formatted by removing missing or ambiguous entries. For each pollinator family, we filtered the dataset accordingly and then applied the *distinct()* function from the dplyr package to identify unique publisher names. Subsequently, the *n_distinct()* function was used to count the number of distinct publishers contributing records for each family.

## 3. Results

### 3.1. Spatial Bias

Table 1 summarizes the pollinator dataset used in this study, presenting the initial number of occurrence records and the unique records retained after removing duplicates for each pollinator family. Across the seven bee families, a substantial portion of the data—approximately 81.4% on average—was removed due to duplication. For example, Halictidae showed the highest removal rate at 89.3%, indicating extensive redundancy in its records, while Apidae had about 76.2% duplicates eliminated. The hoverfly family Syrphidae similarly experienced a high duplicate removal rate of 77.2%, reflecting the need for careful data cleaning.

Among the six butterfly families analyzed, the average duplicate removal rate was somewhat lower, at around 65.4%, but was still significant. Notably, Nymphalidae exhibited the highest duplication, with over 91% of records removed, suggesting a large number of repeated occurrences in the original dataset. In contrast, Papilionidae had minimal duplications, with only about 3.9% of records removed, indicating relatively cleaner initial data.

**Figure 1:**
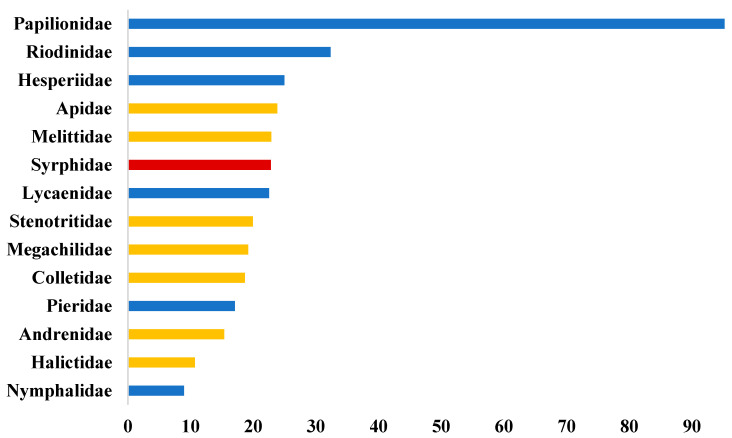
Percentage of unique pollinator records after duplicate removal, shown for each pollinator family grouped by pollinator type (bees, hoverflies, butterflies). The horizontal bars represent the proportion of unique records relative to the initial total records for each family. The bar color separates pollinator groups for clarity.

Figure 1 complements this table by visually representing the percentage of unique records retained for each family after duplicate removal, grouped by pollinator group (bees, hoverflies, butterflies). The figure clearly illustrates the variability in data quality and duplication levels across families, emphasizing the importance of rigorous data refinement to ensure reliable downstream analyses.

### 3.2. Pollinator Occurrence Mapping

We assessed the occurrence bias at the continental scale by extracting the maximum occurrence data of bees, butterflies, and hoverflies per continent. For bees, the highest occurrence data were observed in Europe (3801) and North America (3385), suggesting either greater sampling efforts or naturally higher bee diversity. In contrast, Africa (393) and South America (420) exhibited the lowest occurrence data, which could be due to lower sampling intensity or naturally lower diversity in the dataset. Similarly, for butterflies, Europe had an extremely high occurrence data (32,273), suggesting either strong spatial sampling bias or genuinely higher butterfly diversity, while Asia (5557) and North America (7095) also showed high values, reinforcing significant sampling efforts. Africa (877) and South America (1320) again had the lowest values, indicating potential under-sampling in these regions. Hoverflies followed a similar pattern, with Europe (1494) and North America (1109) displaying the highest occurrence data, while Africa (119) and South America (61) had the lowest values, suggesting a major sampling gap in these regions.

**Figure 2:**
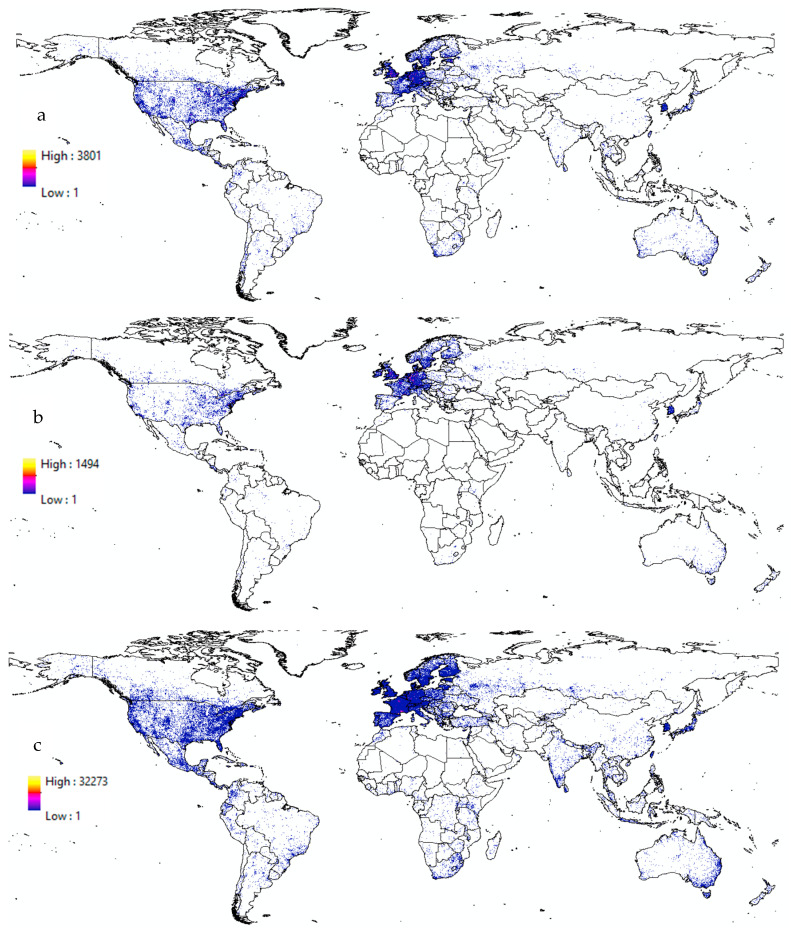
Global occurrence maps of pollinators. (**a**) Bees, (**b**) hoverflies, and (**c**) butterflies.

At the country level, our analysis revealed that the top five countries with the highest occurrence data for bees were the United States with 342,435 records, Germany with 121,076 records, the United Kingdom with 113,356 records, Sweden with 67,117 records, and Canada with 55,718 records. For hoverflies, the leading countries were Germany with 75,896 records, the United States with 68,253 records, the United Kingdom with 43,427 records, France with 38,996 records, and Sweden with 28,755 records. For butterflies, the top countries were France with 829,192 records, the United States with 715,098 records, Germany with 430,989 records, Sweden with 352,442 records, and the United Kingdom with 205,048 records. Figure 2 depicts global occurrence maps of pollinators, illustrating the distribution patterns of bees (a), hoverflies (b), and butterflies (c). The maps highlight a distinct spatial bias in insect pollinator data, concentrating heavily on the USA, European nations, and select countries such as South Korea, Japan, and Australia. Conversely, significant portions of the world, notably northern Africa and central Asia, exhibit sparse sampling, indicating areas where pollinator data are notably lacking.

### 3.3. Pollinator Richness Analysis

**Figure 3:**
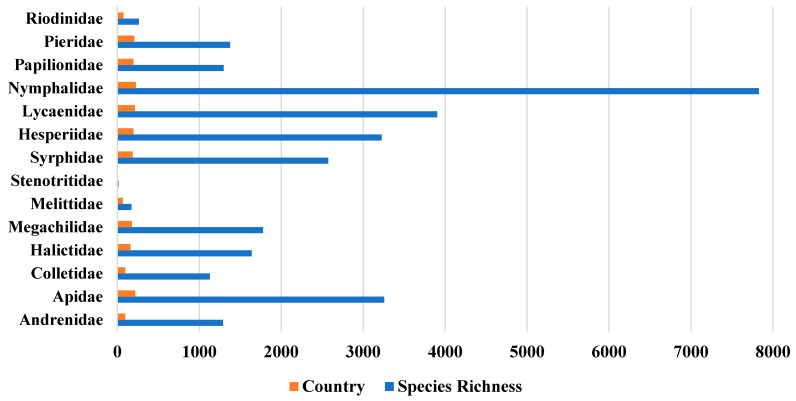
Species richness and geographic distribution of pollinator families. The blue bars show the number of unique species recorded per family, while the red ones display the number of countries in which each family was observed.

Table 1 also summarizes species richness and geographic distribution across major pollinator families, including bees, hoverflies, and butterflies. Among bees, Apidae exhibits the highest species richness (3256 species), followed by Megachilidae (1779) and Halictidae (1640), while Stenotritidae shows the lowest (16 species). The hoverfly family Syrphidae contains 2574 species. Among butterflies, Nymphalidae is the most diverse with 7829 species, followed by Lycaenidae (3904) and Hesperiidae (3226), while Riodinidae has the fewest (264 species). The table also provides the number of countries in which each family has been recorded. Apidae and Nymphalidae show the broadest geographic coverage, with presence in 220 and 226 countries, respectively. Other widely distributed families include Lycaenidae (214), Pieridae (208), Syrphidae (189), and Megachilidae (178), whereas Stenotritidae is reported from only one country. Figure 3 visualizes these patterns by displaying species richness (left panel) and number of countries (right panel) per pollinator family, offering a clearer comparison of both taxonomic and geographic diversity across insect groups.

### 3.4. Temporal Trends Analysis

**Figure 4:**
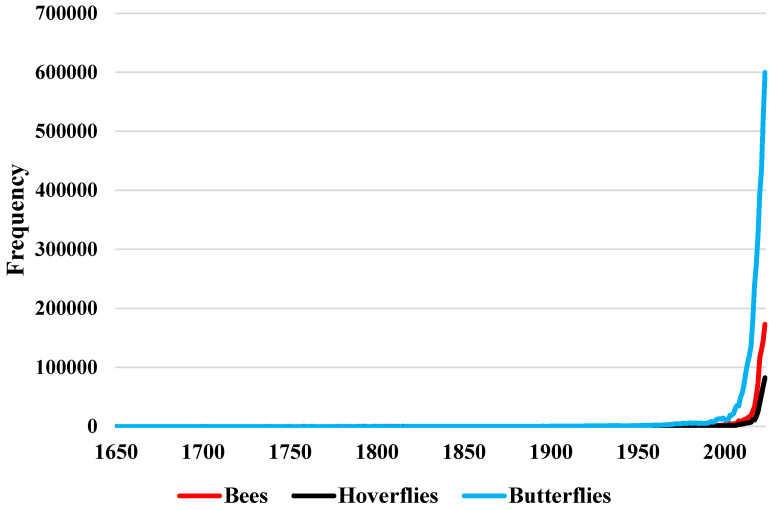
Temporal frequency of pollinator occurrence data.

According to Table 1, Apidae has data collected over the longest period, with 184 years of occurrence records, highlighting the extensive temporal coverage of this family. Andrenidae follows closely with 175 years, indicating robust historical data collection. Halictidae and Megachilidae both have 168 and 162 years, respectively, showing substantial long-term data availability. Colletidae contributes 162 years of data, while Melittidae has 154 years, and Stenotritidae has 31 years. Among hoverflies, Syrphidae exhibits the longest data collection period with 189 years, underscoring comprehensive historical records for this family. For butterflies, Lycaenidae and Nymphalidae both have 200 years of data, indicating extensive temporal coverage and data continuity. Pieridae follows with 185 years, while Hesperiidae and Papilionidae have 180 years each. Riodinidae has 155 years of occurrence data collected, demonstrating the temporal breadth of data availability across butterfly families. Figure 4 shows the temporal frequency of pollinator occurrence data, broken down into bees, hoverflies, and butterflies. The figures and supplementary data reveal that 80% of the occurrence data for bees and butterflies has been recorded in the GBIF database after 2022, while for hoverflies, this threshold is reached after 2023.

### 3.5. Publisher Numbers

Among the bee families, Apidae stands out with the highest number of data publishers (292), indicating strong research collaboration and extensive data sharing within this family. This is followed by Halictidae with 168 publishers and Megachilidae with 158, reflecting substantial but comparatively lower engagement. Other bee families such as Andrenidae and Colletidae also show notable contributions with 134 and 136 publishers, respectively, while Melittidae and Stenotritidae have fewer publishers at 128 and 0 (unreported), respectively.

In the hoverfly group, Syrphidae—the only family listed—has 191 publishers, underscoring a solid but more concentrated scientific interest and collaboration compared with the bees. Within the butterflies, Nymphalidae leads by a considerable margin with 360 publishers, reflecting widespread scientific engagement and data collection efforts. This is followed by Hesperiidae (262 publishers) and Pieridae (258 publishers), which also show strong participation in biodiversity data sharing. Other butterfly families such as Papilionidae, Lycaenidae, and Riodinidae have 245, 229, and 90 publishers, respectively, indicating a broad but variable level of contribution.

Across all three pollinator groups, the top data contributors highlight an impressive international collaboration network. *iNaturalist.org* emerges as the largest single source of records, providing 473,401 bee records, 159,489 hoverfly records, and 1,385,687 butterfly records, demonstrating its pivotal role in citizen science and biodiversity monitoring worldwide. Other significant contributors include *Observation.org* and the *Biological Records Centre*, which primarily support bee data collection, *SLU ArtDatabanken* as a key contributor for hoverfly records, and *UMS PatriNat* for butterfly data.

## 4. Discussion

This study evaluated the global coverage and data quality of pollinator occurrence records from GBIF for bees, hoverflies, and butterflies, focusing on taxonomic richness, geographic distribution, and temporal trends. While the volume of raw data is substantial—over 38 million records across the 3 groups—our findings reveal that a large proportion of these records were duplicates, particularly for bees (81.4%) and hoverflies (77.2%). This aligns with earlier concerns about data redundancy in GBIF [33,34] and highlights the importance of rigorous preprocessing before conducting biodiversity assessments or species distribution modeling.

The relatively high coverage of butterfly species (96.7%) in GBIF compared with bees (45.4%) and hoverflies (42.9%) reflects known taxonomic and reporting biases [13,14,35]. Butterflies are more charismatic and frequently observed, which likely contributes to more complete data. In contrast, certain bee families (e.g., Stenotritidae) and hoverflies remain significantly underrepresented, suggesting that these taxa are less accessible to both researchers and citizen scientists.

These biases are not only taxonomic but also spatial and temporal. Most pollinator data originate from a few well-represented countries—primarily the United States, Germany, the United Kingdom, and Sweden—mirroring patterns reported by Gilman, King, Peterson, Chavan, and Hahn [11] and Garcia-Rosello, Gonzalez-Dacosta, Guisande, and Lobo [14]. Large geographic gaps remain, particularly across Africa, Asia, and Oceania, limiting our understanding of pollinator diversity and trends in these regions. Rocha-Ortega, Rodriguez, and Córdoba-Aguilar [13] also emphasized such disparities, noting vast differences in data density between countries like Sweden and France. As a result, the global picture painted by GBIF is heavily skewed toward regions with robust biodiversity infrastructure and citizen science engagement.

From a temporal perspective, most occurrence records were collected only recently—after 2022 for bees and butterflies, and after 2023 for hoverflies—suggesting a recent surge in interest and reporting. This increase aligns with the global awareness of pollinator decline and the expansion of citizen science platforms like iNaturalist [36,37,38]. However, this trend introduces a potential mismatch when paired with environmental predictors such as WorldClim, which represents climate conditions from 1970 to 2000. As noted by Rahimi et al. [39] and Rahimi and Jung [23], using outdated climatic layers with contemporary occurrence data can introduce artifacts or can misinterpret ecological niches. Therefore, future species distribution model (SDM) studies should prioritize using updated climate data or incorporate dynamic modeling approaches to account for environmental shifts.

One critical takeaway from this study is the role of data publishers in shaping biodiversity knowledge. iNaturalist.org emerged as the top source of pollinator occurrence data across all three groups, followed by Observation.org and regional institutions such as the Biological Records Centre (UK) and UMS PatriNat (France). The dominance of iNaturalist is consistent with its rise as a global platform for citizen science, facilitating massive community-verified contributions [40,41]. Yet this centralization also raises concerns over data consistency, validation, and regional representativeness, especially when certain regions or taxa are underrepresented.

To address the biases identified in global pollinator datasets, several strategies can be implemented. Researchers should acknowledge data limitations and incorporate bias-mitigation techniques such as spatial thinning, rarefaction, or weighted modeling to reduce distortion in analyses. Expanding data collection in underrepresented regions and for less-studied taxa is also crucial and can be achieved through internationally coordinated initiatives and collaborative funding. Data aggregators like GBIF can improve dataset reliability by identifying and filtering duplicate records and by providing users with tools to assess and correct for bias at the point of data download [42]. Additionally, strengthening community participation—especially through citizen science platforms and regional biodiversity monitoring—can help close data gaps and promote more balanced and inclusive biodiversity documentation [33].

## 5. Conclusions

This study analyzed GBIF pollinator occurrence data across 14 families of bees, hoverflies, and butterflies, revealing high levels of duplicate records and a strong temporal bias toward recent years, particularly post-2022—a trend that likely reflects growing global concern over pollinator declines and ecosystem health. While countries such as the U.S., Germany, the U.K., and Sweden contribute the most data, large geographic and taxonomic gaps remain. Butterfly records were the most complete, covering nearly 97% of known species, with platforms like iNaturalist.org and Observation.org playing key roles in data provision through citizen science. However, working with GBIF pollinator data presents limitations, including potential biases in data collection, underrepresentation of certain regions and taxa, and challenges posed by duplicate records that may affect the comprehensiveness and accuracy of biodiversity analyses. Our findings highlight both the value and the limitations of GBIF data for ecological research. Enhancing data quality by addressing duplication, reducing bias, and expanding coverage in underrepresented areas is essential. Future studies should aim to integrate high-quality occurrence records with recent environmental data to improve species distribution models and support more accurate climate-informed conservation planning.

## Figures and Tables

**Table 1:** Summary of pollinator dataset, including initial records, unique records, species richness per family, species per country, records per year, and unique publisher numbers.

	Family	Initial Records	Unique	Species	Country	Year	Publisher
**Bees**							
	Andrenidae	341,269	52,471	1292	99	175	134
	Apidae	3,117,038	742,649	3256	220	184	292
	Colletidae	155,654	29,060	1129	98	162	136
	Halictidae	917,318	98,120	1640	161	168	168
	Megachilidae	303,249	58,157	1779	178	162	158
	Melittidae	86,165	19,713	174	68	154	128
	Stenotritidae	1697	338	16	1	31	-
**Hoverflies**							
	Syrphidae	1,703,131	388,326	2574	189	189	191
**Butterflies**							
	Hesperiidae	1,781,922	444,716	3226	197	180	262
	Lycaenidae	3,214,750	724,376	3904	214	200	229
	Nymphalidae	22,568,055	2,016,417	7829	226	200	360
	Papilionidae	385,943	370,951	1297	198	180	245
	Pieridae	3,716,986	635,040	1375	208	185	258
	Riodinidae	33,040	10,691	264	76	155	90

## Data Availability

The original contributions presented in this study are included in the article/Supplementary Material. Further inquiries can be directed to the corresponding author.

## Supplementary Materials

The following supporting information can be downloaded at: https://github.com/ehsanrahimi666/GBIF.git (accessed on 10 June 2025).
